# Supplementary material for: SARS-CoV-2 shifting transmission dynamics and hidden reservoirs potentially limit efficacy of public health interventions in Italy
Source: Commun Biol. 2021 Apr 21;4:489. doi: 10.1038/s42003-021-02025-0 (PMC8060392; doi:10.1038/s42003-021-02025-0)
Supplement: Supplementary file 1 — Supplementary Information [file 42003_2021_2025_MOESM1_ESM.pdf]

SUPPLEMENTARY APPENDIX

SARS-CoV-2 shifting transmission dynamics and hidden reservoirs potentially limit efficacy of public health interventions in Italy

Marta Giovanetti[1,2,3]*, Eleonora Cella[4]*, Francesca Benedetti[5]*, Brittany Rife Magalis[6]*, Vagner Fonseca[2,7,8], Silvia Fabris[3], Giovanni Campisi[9], Alessandra Ciccozzi[3], Silvia Angeletti[10], Alessandra Borsetti[11], Vittoradolfo Tambone[12], Caterina Sagnelli[13], Stefano Pascarella[14], Alberto Riva[15], Giancarlo Ceccarelli[16], Alessandro Marcello[17], Taj Azarian[4], Eduan Wilkinson[7], Tulio de Oliveira[7], Luiz Carlos Junior Alcantara[1,2], Roberto Cauda[18], Arnaldo Caruso[9], Natalie E Dean[19], Cameron Browne[20], Jose Lourenco[21], Marco Salemi[6^], Davide Zella[5^], Massimo Ciccozzi[3^]

[1]Laboratório de Flavivírus, Instituto Oswaldo Cruz, Fundação Oswaldo Cruz, Rio de Janeiro, Brazil; [2]Laboratório de Genética Celular e Molecular, ICB, Universidade Federal de Minas Gerais, Belo Horizonte, Minas Gerais, Brazil; [3]Medical Statistic and Molecular Epidemiology Unit, University of Biomedical Campus, Rome, Italy; [4]Burnett School of Biomedical Sciences, University of Central Florida, Orlando (Florida, USA); [5]Institute of Human Virology and Global Virus Network Center, Department of Biochemistry and Molecular Biology, University of Maryland School of Medicine, Baltimore, MD 21201, USA; [6]Emerging Pathogens Institute & Department of Pathology, College of Medicine, University of Florida, Gainesville, FL 32610, USA; [7]KwaZulu-Natal Research Innovation and Sequencing Platform (KRISP), School of Laboratory Medicine and Medical Sciences, College of Health Sciences, University of KwaZulu-Natal, Durban, South Africa; [8]Coordenação Geral dos Laboratórios de Saúde Pública/Secretaria de Vigilância em Saúde, Ministério da Saúde, (CGLAB/SVS-MS) Brasília, Distrito Federal 70719-040, Brazil; [9]Department of Molecular and Translational Medicine, Section of Microbiology, University of Brescia, Brescia, Italy; [10]Unit of Clinical Laboratory Science, University Campus Bio-Medico of Rome, Rome, Italy; [11]National HIV/AIDS Researh Center, Istituto Superiore di Sanità, Rome, Italy; [12]Anthropology, and Applied Ethics, Campus Bio-Medico University, Rome, Italy; [13]Department of Mental Health and Public Medicine, University of Campania "Luigi Vanvitelli", Naples, Italy; [14]Department of Biochemical Sciences "A. Rossi Fanelli", University of Rome “La Sapienza”, Rome, Italy; [15]ICBR, University of Florida,

Gainesville, FL 32610, USA; [16] Department of Public Health and Infectious Diseases, Policlinico Umberto I Università 'Sapienza', Rome; [17]Laboratory of Molecular Virology, International Centre for Genetic Engineering and Biotechnology (ICGEB), Trieste, Italy; [18]Department Infectious Diseases, – Fondazione Policlinico Universitario "A. Gemelli" IRCCS, Rome, Italy; [19] Department of Epidemiology, College of Public Health and Health Professions, University of Florida, Gainesville, FL 32610, USA; [20] Department of Mathematics, University of Lafayette, LA, USA; [21]Department of Zoology, University of Oxford, Oxford OX1 3PS, UK.

*These authors contributed equally to this article.

^Correspondence and requests for materials should be addressed to m.ciccozzi@unicampus.it;

Dzella@ihv.umaryland.edu; salemi@pathology.ufl.edu

Supplementary Figure 1

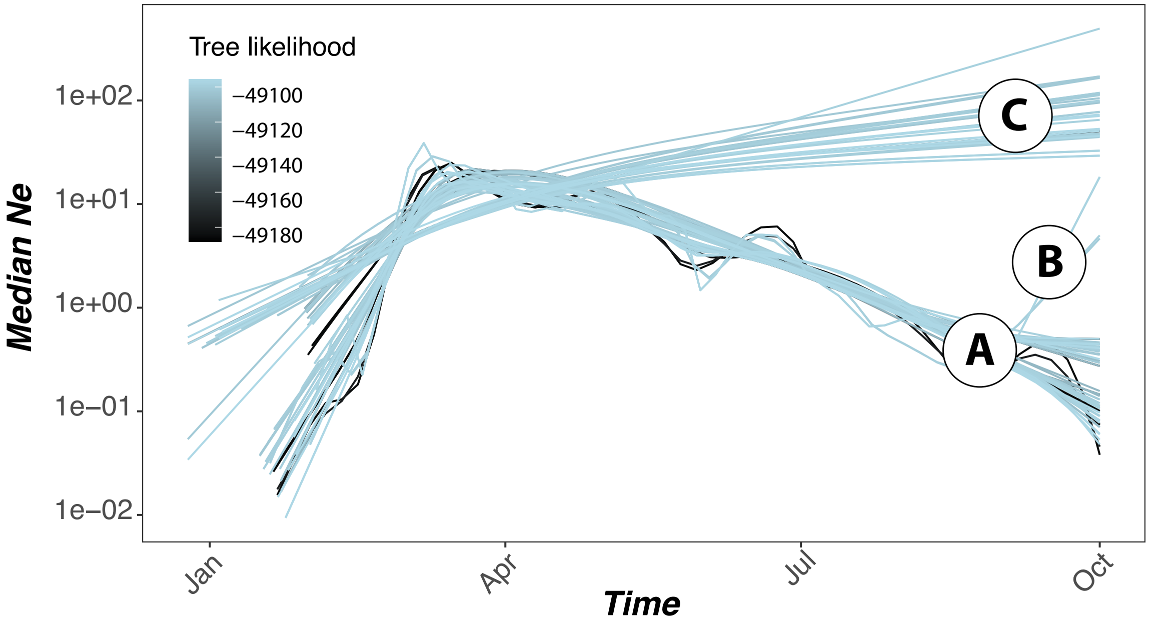

Supplementary Figure 1. Estimates of the viral effective population size (Ne) in the Italian epidemic. Estimates of the viral effective population size (Ne) using a sample of 100 phylogenetic trees with the highest log-likelihood values. Encircled letters (A, B and C) label the three major patterns inferred from collection of trees with the highest likelihood values.

Supplementary Figure 2

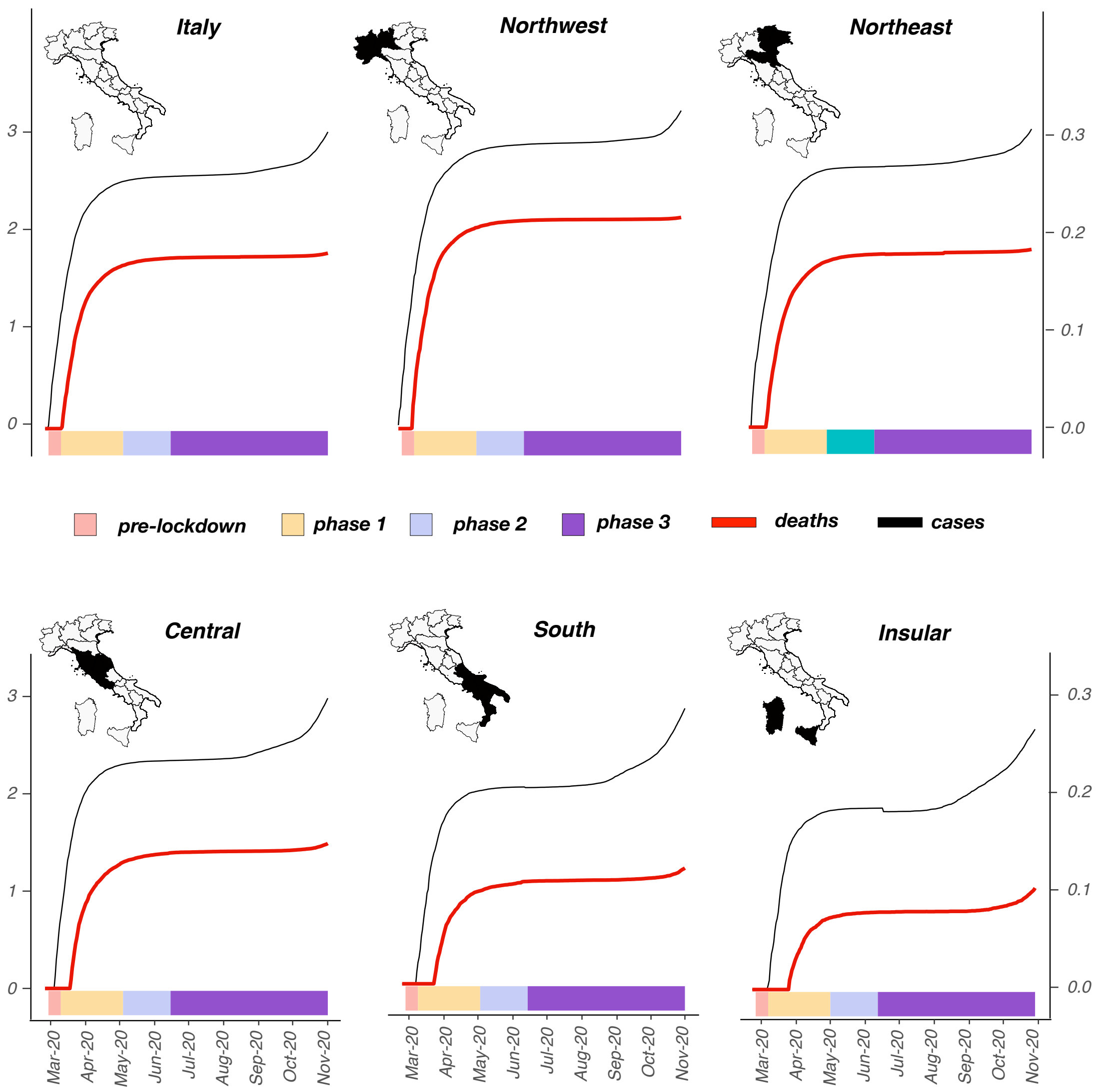

Supplementary Figure 2. Spatial and temporal distribution of SARS-CoV-2 trough Italian regions. Italian regions were aggregated into five macro regions (NUTS, Nomenclature of territorial units for statistics): Northeast, Northwest, Central, South and Insular. The left-hand Y-axis (left) represents incidence (cases per 100K population, black curve) of COVID-19, while the secondary Y-axis (right) represents the number of deaths (red curve) related to COVID-19 through the country. Y-axis numbers are represented as log10 for visual purposes. Colours at the bottom represent the epidemic phases in Italy: pre-lockdown (early transmission) in light red, phase 1 in yellow, phase 2 in light violet and phase 3 in purple. Maps of Italy were superimposed to showing to exact location of each region: Northwest; Northeast; Central; South and Insular.

Supplementary Figure 3

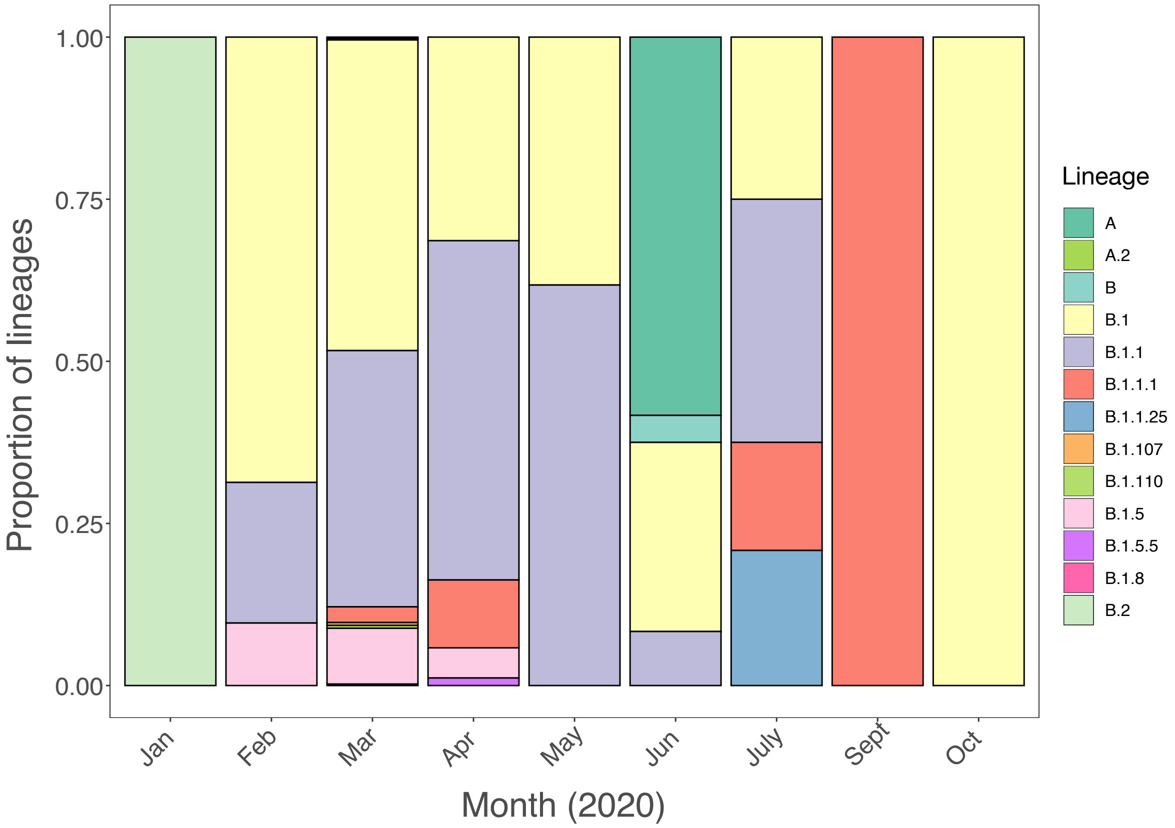

Supplementary Figure 3. Frequency and distribution of SARS-CoV-2 lineages in Italy over time.

Supplementary Figure 4

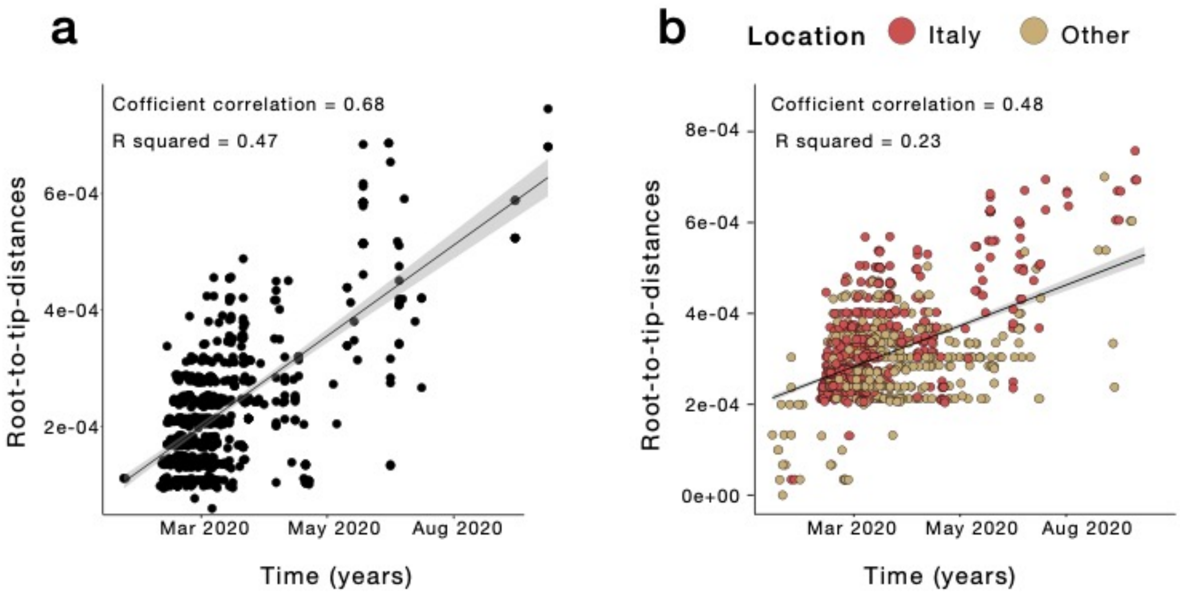

Supplementary Figure 4. Analysis of temporal structure. **a.** Root-to-tip genetic divergence of Italian sequences against time of sampling. **b.** Root-to-tip genetic divergence for the whole dataset (Italian strains + reference sequences) against time of sampling.

Supplementary Figure 5

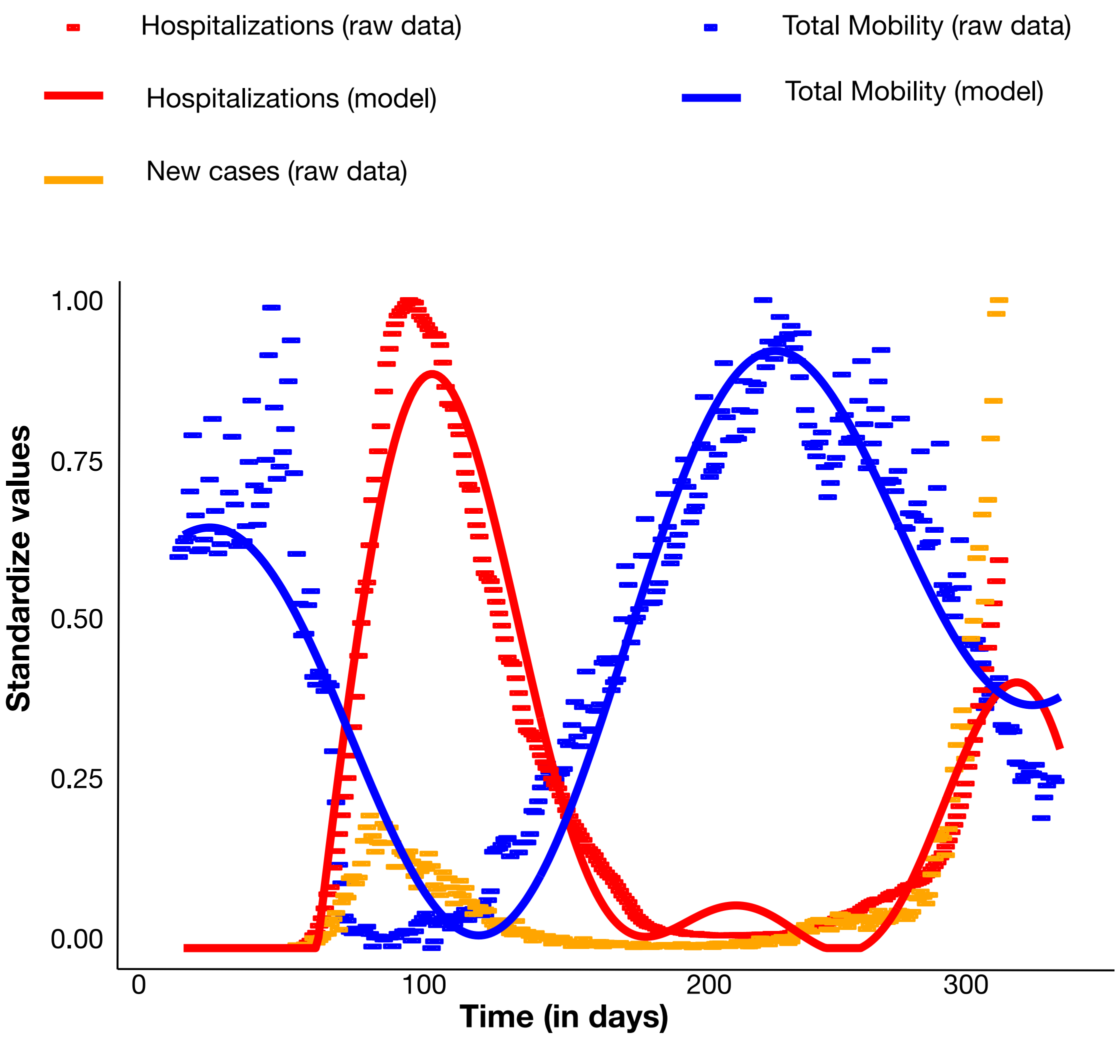

Supplementary Figure 5. Empirical observations (points) and chosen model (lines) for hospitalization rates (red) and number of mobile individuals (blue) over time. Hospitalization rates represent the number hospitalized per positive case, whereas mobility represent the number of individuals utilizing walking, as well as public and personal modes of transportation, as primary means of mobility (blue). Values were standardized for comparison. Empirically observed number of new infections (standardized) are also shown for comparison in orange.
