# Supplementary material for: SARS-CoV-2 shifting transmission dynamics and hidden reservoirs potentially limit efficacy of public health interventions in Italy
Source: Commun Biol. 2021 Apr 21;4:489. doi: 10.1038/s42003-021-02025-0 (PMC8060392; doi:10.1038/s42003-021-02025-0)
Supplement: Supplementary file 2 — Description of Additional Supplementary Files [file 42003_2021_2025_MOESM2_ESM.pdf]

Description of Additional Supplementary Files

File Name: Supplementary Data 1

Description: Metadata of the Italian strains analyzed in this study

File Name: Supplementary Data 2

Description: Metadata of all the reference strains used in this study

File Name: Supplementary Data 3

Description: Genome data by the Authors from the Originating laboratories responsible for obtaining the specimens, as well as the Submitting laboratories generated and shared via GISAID, on which this research is based.
