# Supplementary material for: SARS-CoV-2 shifting transmission dynamics and hidden reservoirs potentially limit efficacy of public health interventions in Italy
Source: Commun Biol. 2021 Apr 21;4:489. doi: 10.1038/s42003-021-02025-0 (PMC8060392; doi:10.1038/s42003-021-02025-0)
Supplement: Supplementary file 6 — Reporting Summary [file 42003_2021_2025_MOESM6_ESM.pdf]

nature research

Corresponding author(s): Marco Salemi

Last updated by author(s): Feb 23, 2021

Reporting Summary

Nature Research wishes to improve the reproducibility of the work that we publish. This form provides structure for consistency and transparency in reporting. For further information on Nature Research policies, see our Editorial Policies and the Editorial Policy Checklist.

Statistics

For all statistical analyses, confirm that the following items are present in the figure legend, table legend, main text, or Methods section.

n/a	Confirmed	
☐	☒	The exact sample size (*n*) for each experimental group/condition, given as a discrete number and unit of measurement
☒	☐	A statement on whether measurements were taken from distinct samples or whether the same sample was measured repeatedly
☐	☒	The statistical test(s) used AND whether they are one- or two-sided<br>*Only common tests should be described solely by name; describe more complex techniques in the Methods section.*
☐	☒	A description of all covariates tested
☐	☒	A description of any assumptions or corrections, such as tests of normality and adjustment for multiple comparisons
☐	☒	A full description of the statistical parameters including central tendency (e.g. means) or other basic estimates (e.g. regression coefficient) AND variation (e.g. standard deviation) or associated estimates of uncertainty (e.g. confidence intervals)
☐	☒	For null hypothesis testing, the test statistic (e.g. *F*, *t*, *r*) with confidence intervals, effect sizes, degrees of freedom and *P* value noted<br>*Give P values as exact values whenever suitable.*
☐	☒	For Bayesian analysis, information on the choice of priors and Markov chain Monte Carlo settings
☒	☐	For hierarchical and complex designs, identification of the appropriate level for tests and full reporting of outcomes
☐	☒	Estimates of effect sizes (e.g. Cohen's *d*, Pearson's *r*), indicating how they were calculated

Our web collection on statistics for biologists contains articles on many of the points above.

Software and code

Policy information about availability of computer code

Data collection: no software was used.

Data analysis:
mask_alignment_using_vcf.py
MAFFT v. 7.475
Aliview v.1.26
pangolin v.2.0.8
IQ-TREE v.1.6.10
TempEst v1.5.3
treedater v. 0.5.0
R v.3.6.0
Phylopart v.2
phytools v.0.7-70
ape v.5.4-1
dplyr v.1.0.2
purr v.0.3.4
rlist v.0.4.6.1
tidytree v.0.3.3
ggplot2 v.3.3.2
data.table v.1.13.6
reshape2 v.1.4.4
lubridate v.1.7.9.2
ggtree v.1.4.11
tidyr v.1.1.2

parallel v.3.6.2
EpiEstim R v.2.2-4
nosoi v.1.0.3

For manuscripts utilizing custom algorithms or software that are central to the research but not yet described in published literature, software must be made available to editors and reviewers. We strongly encourage code deposition in a community repository (e.g. GitHub). See the Nature Research guidelines for submitting code & software for further information.

Data

Policy information about availability of data

All manuscripts must include a data availability statement. This statement should provide the following information, where applicable:

- Accession codes, unique identifiers, or web links for publicly available datasets
- A list of figures that have associated raw data
- A description of any restrictions on data availability

Sequence and epidemiology raw data utilized, generated or analyzed during these studies are available from the authors upon request (including sequence alignment and R scripts for the phylodynamic analyses).

Field-specific reporting

Please select the one below that is the best fit for your research. If you are not sure, read the appropriate sections before making your selection.

☐ Life sciences ☐ Behavioural & social sciences ☒ Ecological, evolutionary & environmental sciences

For a reference copy of the document with all sections, see nature.com/documents/nr-reporting-summary-flat.pdf

Ecological, evolutionary & environmental sciences study design

All studies must disclose on these points even when the disclosure is negative.

Study description	we coupled phylodynamic analysis of viral genetic and epidemiology data, including all currently available SARS-CoV-2 full genome sequences (n=714) from Italian patients, to investigate the interplay between public health intervention and shifting SARS-CoV-2 transmission dynamics. Our work provides a model of explanation for the observed oscillation between times of relatively stable epidemic recession and dramatic resurgence, as it is currently being observed in several other countries worldwide, especially in countries (such as UK, France and Germany) that have adopted aggressive lockdown measures.
Research sample	we dowloaded all Italian full-length viral genomes available on GISAID (https://www.gisaid.org/) (n=714) up to October 31th 2020. Each Italian sequence was used in a local alignment (BLAST) search for the most (genetically) similar non-Italian sequence in the GISAID database as of Oct 31st, 2020, and linked to two reference sequences including the best match (highest E-value) with a date occurring within one month following, as well as one month prior to the sampling date of the Italian sequence (although, in some cases, only a single non-Italian reference sequence fulfilling one of the inclusion criteria could be found for multiple Italian query sequences). After removing duplicate sequences and masking mutations potentially associated with common sequencing errors, using a vcf filter, a final dataset of 1,421 reference sequences was assembled.
Sampling strategy	all Italian full-length viral genomes available on GISAID (https://www.gisaid.org/) (n=714) up to October 31th 2020 were downloaded. Each Italian sequence was used in a local alignment (BLAST) search for the most (genetically) similar non-Italian sequence in the GISAID database as of Oct 31st, 2020, and linked to two reference sequences including the best match (highest E-value) with a date occurring within one month following, as well as one month prior to the sampling date of the Italian sequence (although, in some cases, only a single non-Italian reference sequence fulfilling one of the inclusion criteria could be found for multiple Italian query sequences).
Data collection	all Italian full-length viral genomes available on GISAID (https://www.gisaid.org/) (n=714) up to October 31th 2020 were downloaded. The data was collected by MG, EC.
Timing and spatial scale	From January 18th to October 31st 2020 (Italy and worldwide).
Data exclusions	partial and low-quality genomes were excluded.
Reproducibility	the attempts to reproduce the analyses were successful.
Randomization	we dowloaded all Italian full-length viral genomes available on GISAID (https://www.gisaid.org/) (n=714) up to October 31th 2020. Each Italian sequence was used in a local alignment (BLAST) search for the most (genetically) similar non-Italian sequence in the GISAID database as of Oct 31st, 2020, and linked to two reference sequences including the best match (highest E-value) with a date occurring within one month following, as well as one month prior to the sampling date of the Italian sequence (although, in some cases, only a single non-Italian reference sequence fulfilling one of the inclusion criteria could be found for multiple Italian query sequences).
Blinding	Not applicable.

Did the study involve field work? ☐ Yes ☒ No

Reporting for specific materials, systems and methods

We require information from authors about some types of materials, experimental systems and methods used in many studies. Here, indicate whether each material, system or method listed is relevant to your study. If you are not sure if a list item applies to your research, read the appropriate section before selecting a response.

Materials & experimental systems

n/a	Involved in the study
☒	☐ Antibodies
☒	☐ Eukaryotic cell lines
☒	☐ Palaeontology and archaeology
☒	☐ Animals and other organisms
☒	☐ Human research participants
☒	☐ Clinical data
☒	☐ Dual use research of concern

Methods

n/a	Involved in the study
☒	☐ ChIP-seq
☒	☐ Flow cytometry
☒	☐ MRI-based neuroimaging
